# A rare case of splenic abscess with septic peritonitis in a German shepherd dog

**DOI:** 10.1186/s12917-014-0201-z

**Published:** 2014-09-04

**Authors:** Ahmed Abdellatif, Charlotte Günther, Christine Peppler, Martin Kramer

**Affiliations:** Department of Veterinary Clinical Science, Clinic for Small Animals (Surgery), Justus-Liebig University, Frankfurter Street 108, Giessen, 35392 Germany; Animal Surgery Department, Assiut University, Assiut, 71515 Egypt

**Keywords:** Spleen, Splenic abscess, Peritonitis, Ultrasonography, Dog

## Abstract

**Background:**

Splenic abscess is a rare disease with only few reports in small-animal practice as well as in human medicine. It has been mostly reported in immunocompromised patients or following penetrating foreign bodies. This report aims to add to the current veterinary literature on recommended diagnostic tools for splenic abscess, as well as to provide follow-up findings after successful surgical treatment.

**Case presentation:**

An 8-year-old male German shepherd dog was admitted to the clinic for evaluation of fever, anorexia, and lethargy for the previous 3 days. During the physical examination, a mass was palpated in the left cranioventral abdomen. Diagnostic imaging including radiography and ultrasonography revealed the presence of a large mass in the spleen and signs of peritonitis. Laboratory tests reflected highly septic pyogranulomatous inflammation and, together with imaging findings, raised a high suspicion of splenic abscess and septic peritonitis. Therapy included complete splenectomy and placement of peritoneal drainage. Bacteriological examination revealed severe infection with *Staphylococcus epidermidis* and *S. pseudintermedius*. Histopathological evaluation of the mass confirmed the diagnosis of splenic abscess.

**Conclusion:**

Early diagnosis of splenic abscess in small animals requires a high level of suspicion based on clinical and ultrasonographic findings. Immediate surgical intervention is preferable and confirms the diagnosis. Total splenectomy remains the most effective therapy. Although there are many predisposing factors for splenic abscess, the true etiology remains obscure.

## Background

The spleen is an effective filter for organisms and particulate matter, and appears very resistant to infection [1]. Splenic abscesses are very rare in dogs and cats as well as in humans [2,3]. The frequency of splenic abscess in humans is supposed to be rising because of increasing numbers of immunocompromised and cancer patients, who are mostly at risk for this disease [3,4]. Splenic abscesses may be associated with other conditions such as torsion of the vascular pedicle, which can compromise vascular supply or drainage of the spleen, resulting in congestion, hypoxia, and necrosis of the splenic parenchyma with potential abscess formation [2]. Additionally, penetrating foreign bodies have been reported as a causative factor in splenic abscess [5], and in one dog, splenic abscess was diagnosed intraoperatively and histopathologically 3 weeks following an abdominal trauma [6]. In dogs, leishmaniasis is the only immunosuppressive condition that has been reported as a strong predisposing factor for abscess formation, and such a case has been reported in a Spanish mastiff dog [7]. In dogs and cats, microorganisms such as fungi, bacteria, and protozoa, and rarely yeast, have been described to result in generalized splenomegaly and focal lesions, which manifest as chronic suppurative splenitis [2,8]. Although the veterinary literature notes secondary septic peritonitis as a consequence of splenic abscess, according to the author’s knowledge, there are no small-animal reports describing both conditions as well as the successful management. This case report describes the appropriate diagnosis and management of a splenic abscess with septic peritonitis in a German shepherd dog.

## Case presentation

An 8-year-old male German shepherd dog weighing 35 kg was presented with fever, anorexia, and lethargy of 3 days’ duration. There was no history of previous medical or surgical treatment. The general examination revealed fever (40.5°C) and tachycardia (140 beats/min). The respiration rate was 30 breaths/min; the pulse quality was moderately strong, and no arrhythmias were auscultated. The abdomen was distended and painful, and a large mass was palpated in the cranioventral abdomen. The ethical and owner approval was not required.

Laboratory tests, including complete blood count (CBC), serum biochemistry panel, electrolyte levels, coagulation profile, and urinalysis were performed. The abnormalities included leukocytosis (54.5 × 10^9^/L; reference range, 5.48–13.74 × 10^9^/L), neutrophilia (47.4 × 10^9^/L; reference range, 2.78–8.73 × 10^9^/L), marked left shift (8.18 × 10^9^/L; reference range, 0–0.5 × 10^9^/L), decreased packed cell volume (PCV) (34%; reference range, 39–56%), hyponatremia (139 mmol/L; reference range, 141–146 mmol/L), hypoalbuminemia (17.5 g/L; reference range, 29.6–37.01 g/L), and increased alkaline phosphatase activity (300 U/L; reference range, 0–30 U/L). The prothrombin time (PT) was prolonged (17.9 s; reference range, 9.85–14.22 s), and the urine specific gravity was 1.008 (reference range, 1.001–1.065).

**Figure 1:**
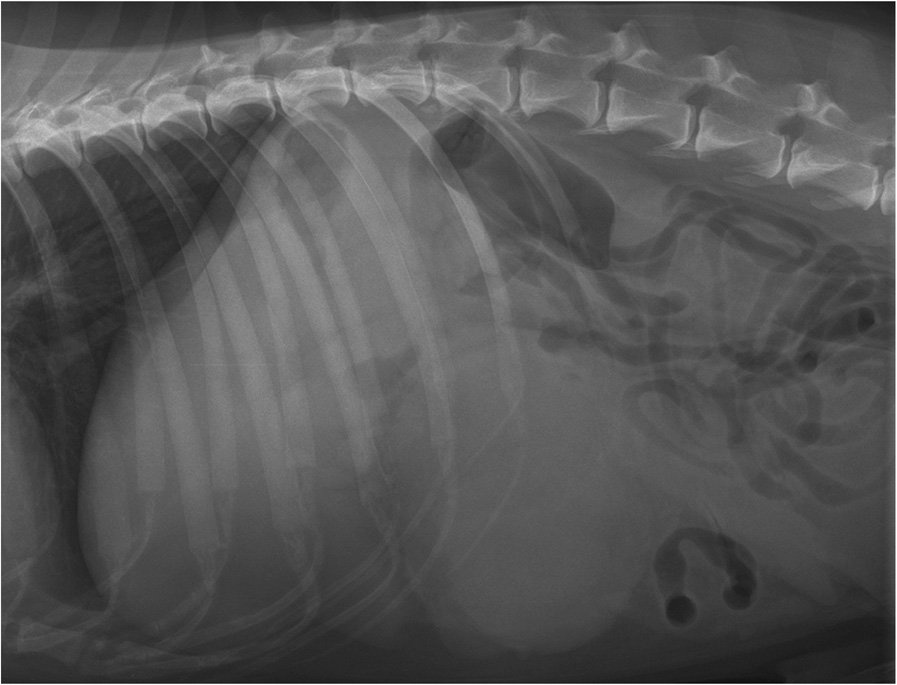
**Lateral radiographic view of the abdominal cavity showing a large soft-tissue density in the cranial part of the abdomen and reduced details.**

**Figure 2:**
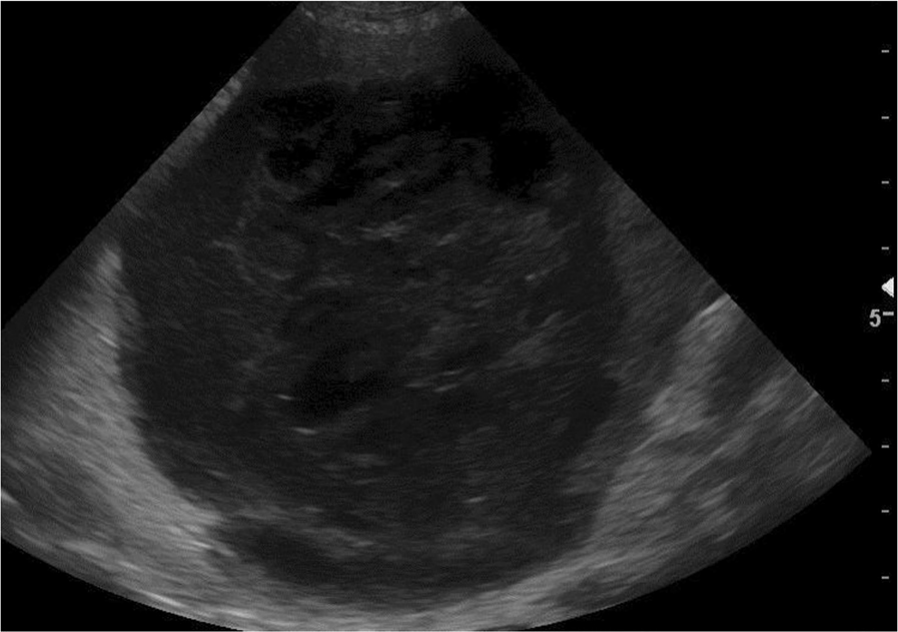
**Sagittal ultrasound image of the left cranial abdomen over the palpated mass demonstrating a 10 cm diameter inhomogeneous swelling continuous with the spleen with hypo- and hyperechogenic areas.** The surrounding tissue is hyperechogenic.

Abdominal radiographs showed a round soft-tissue density, 15 cm in diameter, located in the left cranial part of the abdomen (Figure 1). There was marked loss of serosal contrast, particularly around the mass. Ultrasonographic examination revealed an inhomogeneous enlarged mass 10 × 10 cm in diameter with hypo- and hyperechogenic areas. The mass occupied the cranioventral surface of the spleen, and the surrounding mesentery and adipose tissue appeared blurred and swollen (Figure 2). Mild to moderate peritoneal effusion was seen in the left cranial abdomen. The ultrasound findings of the remaining abdominal organs were unremarkable except for the liver, which was prominent and heterogeneous. The owner declined any further diagnostic procedures such as computed tomography (CT). The animal was directed to exploratory laparotomy after stabilization with a high suspicion of abscess in the spleen.

The dog was stabilized with a “shock dose” of balanced electrolyte solution^a^ (90 mL/kg, IV). Preemptive analgesia was performed with intravenous dipyrone^b^ (40 mg/kg, IV). For premedication and induction of anesthesia, diazepam^c^ (0.5–1.0 mg/kg, IV), L- methadone^d^ (0.2–0.5 mg/kg, IV), and propofol^e^ (2–4 mg/kg, IV) were administered. For maintenance of the anesthesia, a mixture of isoflurane (1.5 vol.%) and oxygen (600–800 mL/ min) was used with a tidal volume of 10–15 mL/kg for inhalation anesthesia. The ventilation rate was controlled (15 mL/kg) with a frequency of 10 breaths/min. Two central vein catheters^f^ were inserted in the left and right jugular veins. Anesthetic monitoring was carried out by means of temperature control, capnography, pulse oximetry, and electrocardiography. Antibiotic therapy consisting of enrofloxacin^g^ (5 mg/kg, IV) and amoxicillin-clavulanic acid^h^ (8.75 mg/kg, SC) was administered to continue the initial preoperative therapy approved by the attending veterinarian [9,10]. Intraoperatively, a single dose of ampicillin^i^ (40 mg/kg, IV) was administered to overcome the septic condition [9].

**Figure 3:**
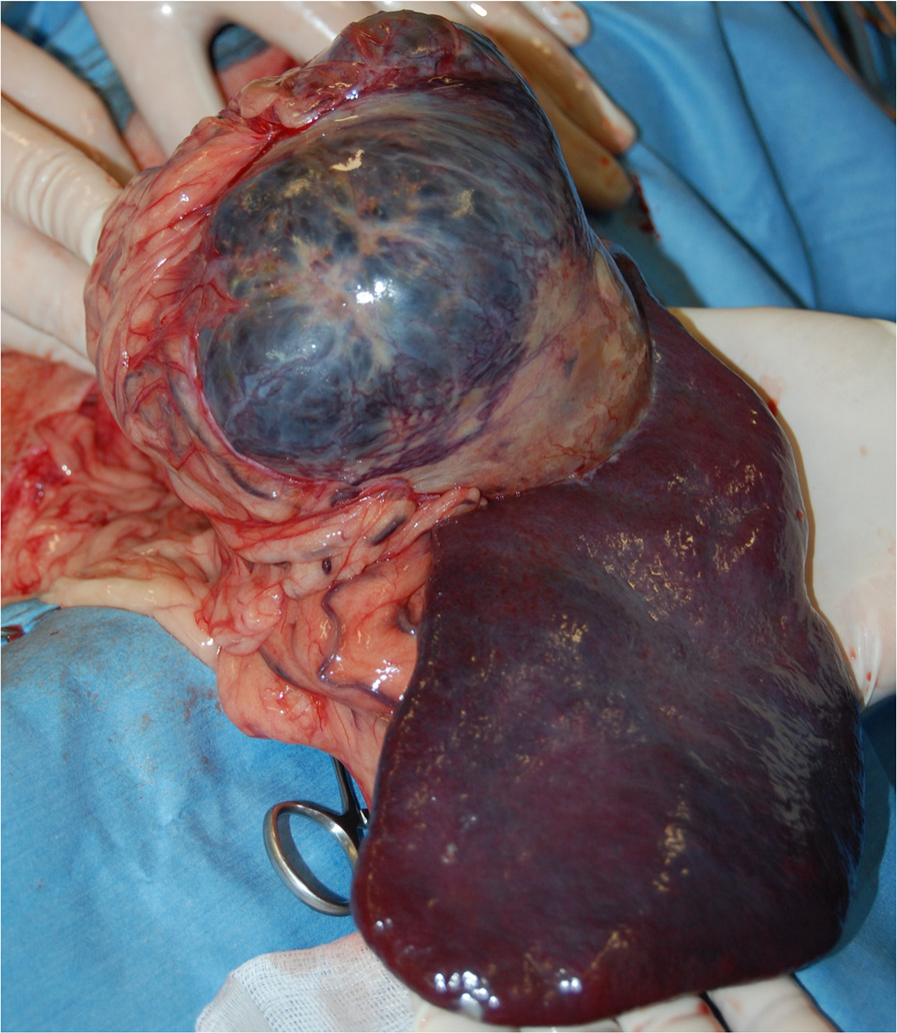
**Intra-operative view of the spleen in this dog, showing the large size of the abscess.**

A celiotomy was performed; a notably musty smell was perceived, and the entire omentum had a yellowish to greenish color. A 10-cm coarse yellowish-white swelling, which occupied the ventral aspect of the spleen, was identified (Figure 3). Diffuse peritonitis and suppurative peritoneal effusion around the swelling were also observed. A swab of the peritoneal cavity was submitted for aerobic and anaerobic bacterial cultures. Total splenectomy using LigaSure^**j**^ was performed. Hilar vessels were sealed as close as possible to the parenchyma. Spleen and liver biopsy samples were sent for histopathologic evaluation. The abdominal cavity was lavaged with 200 mL/kg warm sterile physiological solution. An abdominal Jackson-Pratt drain^k^ was inserted before routine closure of the abdominal cavity. Abdominal lavage with 500 mL of warm sterile physiological solution was performed twice daily through this drain.

**Figure 4:**
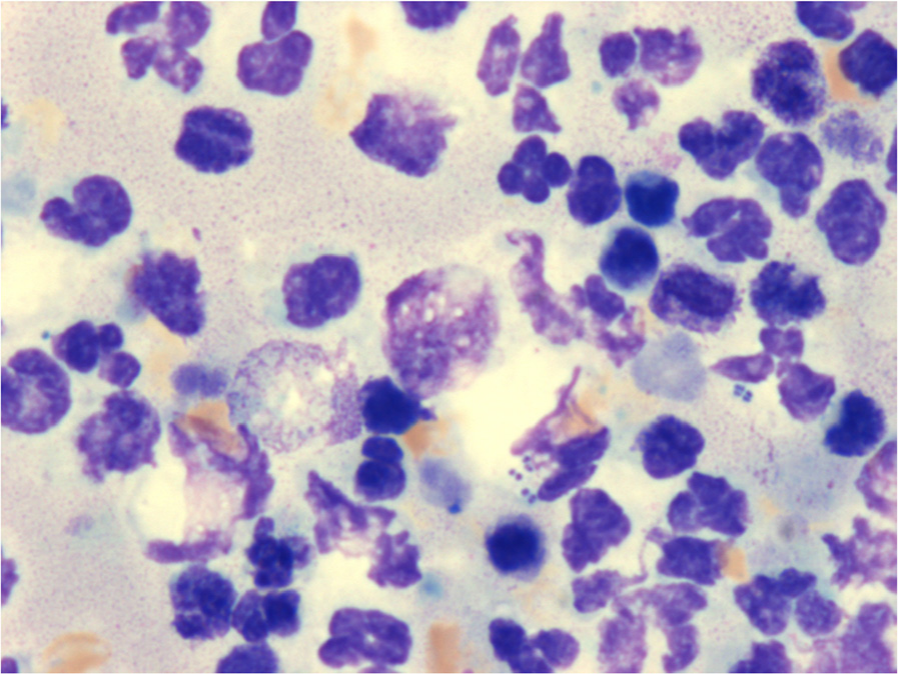
**Cytological image shows severe highly septic pyogranulomatous inflammation with groupings of intra- and extracellular bacteria.**

Culture results from the abdominal cavity demonstrated severe infection with *Staphylococcus epidermidis* and *S. pseudintermedius*. Cytological examination of the spleen demonstrated the presence of masses of neutrophils, few macrophages (with an eccentrically located nucleus with reticulated chromatin), and moderate amounts of cytoplasm-bright basophils. Furthermore, there were numerous intra- and extracellular bacteria arranged in chains (cocci) (Figure 4). Histopathological examination of the spleen and liver biopsy specimens demonstrated the presence of necrotic splenitis as well as multifocal purulent hepatitis and septic peritonitis.

Immediate postoperative treatment included ongoing administration of balanced electrolyte solution together with fresh frozen plasma (7 mL/kg). Postoperative analgesia was provided with Dipyrone (50 mg/kg, IV, q 8 h). For postoperative antibiotic therapy, enrofloxacin (5 mg/kg, IV, q 12 h) and amoxicillin-clavulanic acid ^l^ (20 mg/kg, IV, q 8 h) were continued for 10 days [11]. Central venous pressure and urine production measurements were routinely taken every day and were within the normal parameters.

One day postoperatively, the CBC revealed moderate leukocytosis (39.5 × 10^9^/L), mild neutrophilia (34.8 × 10^9^/L) with marked left-shift bands (4.26 × 10^9^/L), moderate thrombocytopenia (97 × 10^9^/L), and decreased PCV (28%). The blood biochemistry profile showed marked hypoalbuminemia (12.7 g/L) and elevations of the bilirubin (16.72 mmol/L) and alkaline phosphatase (269 U/L) levels. Human albumin^m^ 40 g (200 g/L) and 500 mL whole blood (DEA 1.1 positive, negative crossmatch) were provided. Three days postoperatively, the CBC still showed moderate leukocytosis (41 × 10^9^/L), mild neutrophilia (35.6 × 10^9^/L), moderate left-shift bands (3.26 × 10^9^/L), and slightly increased PCV (30%). The blood biochemistry profile showed a gradual increase in the level of albumin (21.7 g/L). Bilirubin (21.07 mmol/L) and alkaline phosphatase (198 U/L) levels were still elevated. Ultrasonographic examination revealed mild free fluid with signs of peritonitis.

The average amount of fluid drained from the abdomen on postoperative days 1, 2, and 3 was 700 , 300, and 100 mL, respectively. On postoperative day 4, a very small amount of clear fluid was observed, so removal of the abdominal drainage was performed.

Eight days postoperatively, the animal’s general condition was normal. The CBC showed gradual improvement of the numbers of leukocytes (23 × 10^9^/L) and neutrophils (18 × 10^9^/L) and levels of bilirubin (12 mmol/L) and alkaline phosphatase (192 U/L). On the basis of these prior findings, the patient was discharged in a good general condition with ongoing administration of antibiotics and recommendation for further check-up.

After 2 weeks, the animal’s health condition was normal. Laboratory findings revealed unchanged slight leucocytosis, neutrophilia, and elevation of liver enzyme levels. On ultrasonography, there were signs of peritonitis and mild free fluid, which was very little in comparison to previous findings. Two months later, the leucocyte count was normal (12.9 × 10^9^/L) without a neutrophilic left shift. The PCV was 35%, and the albumin (29 g/L), bilirubin (3.3 mmol/L), and liver enzymes had normalized. Ultrasound examination of the abdomen showed no signs of peritonitis or free fluid. Six months postoperatively, the owner reported good health condition of the animal. The offered additional check-up was declined by the owner.

## Discussion

Reports of splenic abscesses are very rare in the veterinary literature. Previous studies in dogs revealed splenic abscess in 2 of 87 dogs with changes in the spleen on pathological examination [5], and in only 4 of 1480 splenic biopsy samples on histopathological examination [8]. In a recent study, splenic abscess was not reported during epidemiological and histopathological examination of 249 splenic masses in dogs [12]. Diffuse splenomegaly caused by splenic abscessation was diagnosed during necropsy of one dog [7]. In cats, splenic abscess was not diagnosed on histopathological examination of 455 samples [8]. However, marked splenitis and septic peritonitis were observed in a cat as a result of splenic foreign body [13].

In humans, splenic abscesses are uncommon with a reported frequency of 0.14– 0.7% in post-mortem studies [14]. However, this uncommon disease has been recently reported more frequently because of advances in imaging modalities and increasing numbers of immunocompromised, trauma, and cancer patients [3]. An additional predisposing factor is abdominal trauma, and splenic abscess has been reported to develop weeks after the trauma event [14]. Metastatic hematogenous infections, splenic infarct, and diabetes have also been shown to be predisposing causes [15].

In the present case, the animal was admitted to the clinic without history of trauma, inherited diseases, or congenital or acquired immunodeficiency. Immunosuppressive medications had not been administered. Splenic abscesses are more likely to occur secondary to conditions that compromise the spleen, such as splenic torsion and thrombosis [16]; however, a single case of a large splenic abscess without any other associated diseases has been documented, which was treated in contrast to our case by partial splenectomy [17]. A possible explanation for the present case is that the animal had sustained a mild undetected trauma that resulted in a splenic hematoma that proceeded as a medium for abscess formation. Such a condition has been mentioned previously in one dog after an abdominal trauma [6].

The clinical presentation of splenic abscess is nonspecific [14]. The vague symptoms of this disease make diagnostic imaging studies very useful for diagnosis [1]. The presented patient showed fever, anorexia, weight loss, and cranial abdominal pain. In addition, a large mass was palpated in the abdomen. Leukocytosis, as found in our case, is the most frequently observed laboratory finding in patients with splenic abscess [18,19]; however, laboratory tests were not remarkable in one dog with splenic abscess following a trauma [6].

Although the use of CT in veterinary medicine is limited by procedure duration, long anesthetic requirement, expense, and availability, recent studies advise the use of CT in the evaluation of splenic masses in stable patients [2]. In human patients, ultrasound plays an important role in the diagnosis of splenic abscess because of its advantages of accuracy, safety, repeatability, low cost [20], and relatively high sensitivity [21,22]. In small animals, there is little or no information detailing the sensitivity of ultrasound for diagnosis of splenic abscess. In the present case, CT was declined by the owner and ultrasonography findings were highly suspicious for splenic abscess. Other diagnostic tools such as abdominocentesis and fine needle aspiration of the spleen were not utilized because of the poor condition of the animal, and immediate surgical intervention was selected.

The most common organisms associated with splenic abscess in humans are aerobic microbes, particularly streptococci and *Escherichia coli* [23]. In animals, other organisms such as *Clostridium* species and *Fusobacterium necrophorum* have also been reported [7]. In our case, severe infection with *Staphylococcus epidermidis* and *S. pseudintermedius* was diagnosed. Purulent splenitis, septic peritonitis, and hepatitis were confirmed on histopathological examination.

Complete splenectomy yields a good prognosis in dogs and cats with splenic abscess if the condition is not complicated by systemic infection [2]. Partial resection of the affected part has also been described [6]. Several human studies have reported a success rate of 100% with splenectomy combined with antimicrobial therapy [15]. Other treatment options in humans include ultrasound or CT-guided percutaneous abscess drainage [15,24]. We proceeded to the classic total splenectomy, which led to rapid and complete resolution of most of the disease-related signs, without any major postoperative complications. Secondary septic peritonitis has been reported to be a consequence of other primary diseases including splenic abscess [7,9]; however, according to our knowledge, there are no case reports describing successful management of both conditions. Diffuse septic peritonitis with evidence of autolysis and putrefaction of most abdominal organs has been associated with a large splenic abscess in one case; however, further diagnostic and treatments options were not performed and the animal was euthanized [7]. In the present case, severe septic peritonitis was observed intraoperatively and confirmed on histopathological examination. Successful management of septic peritonitis was achieved through removal of the primary cause by splenectomy as well as use of an abdominal drainage with ongoing administration of postoperative antibiotics.

## Conclusions

In conclusion, early diagnosis of splenic abscess requires a high degree of suspicion and liberal use of ultrasonographic examination. Rapid surgical intervention is preferable, and total splenectomy remains the most effective therapy. The definitive cause of splenic abscess in this dog remains unknown, although there are many predisposing factors.

## Endnotes

^a^Sterofundin ISO infusion, B. Braun Melsungen AG, Melsungen, Germany.

^b^Metamizol sodium® 500 mg/mL, serum Bernburg, Germany.

^c^Diazepam ratiopharm® 10 mg/2 mL, Ratiopharm, Ulm, Germany.

^d^L-Polamivet® Intervet GmbH, Unterschleissheim, Germany.

^e^Propofol, Narcofol 10 mg/mL; CP-Pharma Handelsgesellschaft GmbH, Burgdorf, Germany.

^f^5-LUMEN-ZVK-SET, ARROWG + ARD BLUE®, ARROW Deutschland GmbH, Germany.

^g^Baytril® 2,5% 50 mL, Bayer HealthCare AG, Leverkusen, Germany.

^h^Synulox RTU 140/35 mg/mL, Pfizer, Berlin, Germany.

^i^Ampicillin ratiopharm® 2.0 g; Ratiopharm, Ulm, Germany.

^j^LigaSure, Covidien Vetoquinol, Elancourt, France.

^k^Jackson-Pratt drain, Oriplast, Neunkirchen-Saar, Germany.

^l^Synulox 250 mg (200 mg/50 mg), Pfizer, Berlin, Germany.

^m^Human albumin, Baxter AG, Unterschleissheim, Germany.

## Abbreviations

WBC: White blood cell
PCV: Packed cell volume
CT: Computed tomography
